# Isolation of a novel strain of *Candida shehatae* for ethanol production at elevated temperature

**DOI:** 10.1186/2193-1801-1-27

**Published:** 2012-10-04

**Authors:** Ayumi Tanimura, Toshihide Nakamura, Itsuki Watanabe, Jun Ogawa, Jun Shima

**Affiliations:** 1 Research Division of Microbial Sciences, Kyoto University, Kitashirakawa Oiwake-Cho, Sakyo-ku, Kyoto, 606-8502 Japan; 2 National Food Research Institute, National Agriculture and Food Research Organization (NARO), 2-1-12 Kannondai, Tsukuba, Ibaraki, 305-8642 Japan; 3 Division of Applied Life Sciences, Graduate School of Agriculture, Kyoto University, Kitashirakawa Oiwake-Cho, Kyoto, Sakyo-ku, 606-8502 Japan

**Keywords:** *Candida shehatae*, Bioethanol production, High-temperature fermentation, Rice straw, SSF

## Abstract

Considering the cost-effectiveness of bioethanol production, there is a need for a yeast strain which can convert glucose and xylose into ethanol at elevated temperatures. We succeeded in isolating a yeast strain, designated strain ATY839, which was capable of ethanolic fermentation at temperatures above those previously reported for yeasts able to ferment both glucose and xylose. Strain ATY839 was capable of producing a substantial amount of ethanol at up to 37°C from 2% glucose or 2% xylose. The results of a phylogenetic analysis suggest that strain ATY839 belongs to *Candida shehatae*. In additional, ethanol production from rice straw by strain ATY839 was examined. Compared with the control strains (*Saccharomyces cerevisiae* NBRC 0224, *Scheffersomyces stipitis* NBRC 10063, and *C. shehatae* ATCC 22984), strain ATY839 produced more ethanol in SSF even at 37°C. The theoretical maximum yield of strain ATY839 was 71.6% at 24 h. Thus, strain ATY839 is considered to be the most tolerant to high temperature of the *C. shehatae* strains.

## Introduction

The efficient use of natural resources for bioethanol production has been explored by several research groups ( [Balat 2011]; [Binod et al. 2010]; [Sarkar et al. 2012]). At present, most bioethanol is produced from food crops such as corn grain or sugar cane ( [Kim & Dale 2004]; [Sanchez & Cardona 2008]); however, the use of starch and sugar for the production of bioethanol competes with crops for food supplies. As an alternative, a lignocellulosic biomass product such as corn stover, corn fiber, rice straw, bagasse or wheat straw could become indispensable resources for bioethanol production. In Japan, for example, approximately 75% of rice straw is not used effectively which could therefore be an abundant feedstock for bioethanol production.

Plant cell walls are composed of three main components: cellulose, hemicellulose, and lignin. To achieve high-efficiency ethanol production, it is desirable to use both the glucose and xylose contained in the cellulose and hemicellulose ( [Kuhad et al. 2011]). However, few types of yeast such as *Scheffersomyces stipitis* (formerly known as *Pichia stipitis*), *Candida shehatae*, and *Spathaspora passalidarum* have been found capable of xylose fermentation ( [Hou 2012]; [Jeffries et al. 2007]; [Prior et al. 1989]); simultaneous utilization of these sugars has been problematic. The most generally used yeast strain in current bioethanol production processes, *Saccharomyces cerevisiae*, can ferment glucose derived from cellulose to ethanol; however, it normally lacks the ability to produce ethanol by fermenting the xylose present in hemicellulose ( [Jeffries & Jin 2004]; [Kuhad et al. 2011]). Thus, there has been extensive exploration to develop yeasts which can produce bioethanol from xylose with a high yield. Toward this end, researchers have tried to genetically improve *S. cerevisiae* and to co-culture two strains. The genetic improvement strategies are founded on the metabolism of wild xylose fermentable yeast, such as *S. stipitis*. For example, xylose reductase and xylitol dehydrogenase genes from *S. stipitis* have been introduced into *S. cerevisiae* in order to make yeast with an improved xylose-fermenting ability ( [Kim et al. 2010]; [Madhavan et al. 2009]). Recombinant yeasts are impractical for industrial use since they require special containment to confine the engineered microorganisms. The use of recombinant yeasts also increases the initial investment and maintenance costs. On the other hand, the co-culture processes are also said to be effective for fermenting both glucose and xylose. The process involves simultaneously utilizing two different yeasts (e.g., *S. cerevisiae* and *S. stipitis*) which are cultured and grown together in the same reactor ( [Fu et al. 2009]; [Yadav et al. 2011]). The main bottleneck in this process so far has been the compatibility of these two strains, as one yeast inhibits the growth of the other ( [Bader et al. 2010]; [Cardona & Sanchez 2007]; [Chen 2011]). Both strategies are being pursued. We therefore consider that a single natural yeast strain is desirable for industrial use.

In addition, fermentation at higher temperatures is desirable for the reduction of cooling costs. This requires the use of yeasts which can produce a good yield of ethanol even at elevated temperatures. In separate hydrolysis and fermentation (SHF), enzymatic hydrolysis and ethanolic fermentation are performed separately. To reduce reactor cooling costs, the temperature difference in the two processes must be reduced. Meanwhile, during simultaneous saccharification and fermentation (SSF), a high temperature is required to accelerate enzymatic hydrolysis ( [Abdel-Banat et al. 2010]; [Banat et al. 1998]; [Fonseca et al. 2008]; [Rodrussamee et al. 2011]). The fermentation reaction temperature is determined by the optimal fermentation temperature of the yeast used in either the SHF or SSF process, with the majority of yeasts growing well in the range of 25°C to 30°C. During industrial bioethanol production, the most widely used yeast is *S. cerevisiae* and its optimal temperature is around 30°C. Consequently, the operating temperatures of glucose and xylose in ethanol conversion systems are fixed at this temperature range. Several researchers have produced bioethanol at high temperatures using thermotolerant yeast strains ( [Hong et al. 2007]; [Nonklang et al. 2008]; [Rajoka et al. 2005]; [Sridhar et al. 2002]); however, ethanol production from only glucose by thermotolerant strains, which were not capable of xylose fermentation to ethanol, have been reported. Considering that xylose is an important sugar in the lignocellulosic biomass (e.g., the xylan content of rice straw can be as high as 20% ( [Roberto et al. 2003])), xylose fermentability would clearly be desirable; to date thermotolerant yeasts have not been used in commercial bioethanol plants. Ethanol production from xylose at elevated temperatures however, as detailed by Banat and Marchant ( [Banat & Marchant 1995]) and by Ishchuk et al. ( [Ishchuk et al. 2008]), resulted in fairly low ethanol productivity. Generally, an increase in temperature leads to a decrease in the rate, yield and/or efficiency of xylose fermentation ( [Rodrussamee et al. 2011]). This suggests it is difficult to identify yeasts suitable for high-yield bioethanol production, which have xylose-fermenting ability and thermotolerance.

In this study, we screened natural yeasts to identify those which can efficiently produce ethanol from xylose and glucose at elevated temperatures. We also examined the ethanol productivity of the selected yeast strain by SSF using rice straw as the feedstock.

## Materials and methods

### Isolation of xylose assimilation yeast strains

Yeast strains were isolated from different natural sources, including flowers, fruits, wood, and soil, obtained from the Kyoto area in Japan (approximately 100 samples in total). The samples were collected in sterilized polypropylene bottles of 15 mL capacity (Becton Dickinson, Franklin Lakes, NJ, USA) and suspended in 10 mL of SX liquid medium (3% xylose and 0.67% YNB without amino acid; Difco, Detroit, MI, USA) containing chloramphenicol at a concentration of 100 μg/mL. The samples were cultivated for 4 days at 30°C in static culture with the lids slightly opened. Aliquots (200 μL) of the culture supernatants were spread onto SX agar medium and incubated aerobically at 30°C for 3 days. Yeasts were purified using single-colony isolation. The yeast strains were routinely maintained on YPD agar plates (2% glucose, 2% peptone [Difco], 1% yeast extract [Difco] and 1.5% agar) and grown at 30°C. YPX medium (4% xylose, 2% peptone [Difco] and 1% yeast extract [Difco]) was used for the screening of xylose-fermenting yeast.

### Selection of xylose-fermenting yeast with thermotolerance

Prior to the fermentation experiments, strain ATY839 was inoculated into 3 mL of YPD medium in test tubes and incubated overnight at 30°C with reciprocal shaking at 150 opm (preculture). The preculture was suspended to 25 mL of synthetic glucose (SG) medium (2% glucose and 0.67% YNB without amino acid) or synthetic xylose (SX) medium (2% xylose and 0.67% YNB without amino acid) in a 50 mL Erlenmeyer flask to a cell optical density of 0.1 at 600 nm (OD_600_) and then cultured at 35°C, 37°C, 38°C, or 39°C for 48 h at 120 rpm. Sugars and ethanol concentrations of the culture supernatants were determined by following the procedures detailed below. Cell growth was determined at OD_600_ using a spectrophotometer. All experiments were performed in triplicate.

### Determination of sugar and ethanol

Sugars and ethanol concentrations were determined using a HPLC (Shimadzu, Kyoto, Japan) equipped with an Aminex Fermentation Monitoring Column (Bio-Rad Laboratories, Hercules, CA, USA) and Micro-Guard Cation H Refill Cartridges with a Standard Cartridge Holder (Bio-Rad Laboratories). Sugars and ethanol were detected using an RID 10A refractive index detector (Shimadzu). The column was kept at 60°C using a CTO 20A column oven (Shimadzu). A sulfuric acid solution (5 mM) was used as the mobile phase at a constant flow rate of 0.6 mL/min. Portions (10 μL) were injected into the HPLC system with a SIL-20A autosampler (Shimadzu), and each run was stopped 14 min after the injection. A Shodex Sugar Column SC1011 (Showa Denko, Tokyo, Japan) was used to measure the glucose and xylose concentrations in SSF hydrolysates: water was used as the mobile phase at a flow rate of 0.6 mL/min and at 70°C. The concentrations of the sugars and ethanol were determined using a standard curve generated by a series of external standards.

### Taxonomic identification of the selected yeast strain

The selected yeast strain was taxonomically identified by 26S rDNA sequencing and the assimilation ability of sugars. The partial 26S rDNA of the strain was amplified by PCR and directly sequenced based on a previous method ( [Kurtzman & Robnett 1998]). The homology of the sequence was determined using the BLAST system of the DNA Data Bank of Japan (DDBJ). The sugar assimilation abilities were evaluated with an API 20C AUX system (BioMerieux, Tokyo, Japan) according to the manufacturer’s instructions.

### Pretreatment of rice straw

The rice straw was pretreated with calcium hydroxide according to the alkali treatment method in CaCCO (calcium capturing by carbonation) process ( [Park et al. 2010]) with some modification. The rice straw employed in this study (cv. Koshihikari) was passed through a high-speed milling machine (PM-2005, Osaka Chemical, Osaka, Japan) and filtered through a 500 μm mesh sieve. The milled rice straw (5 g) was added to a 200 mL Erlenmeyer flask (with a silicone plug) and mixed with calcium hydroxide (1 g) and water (45 g); the flask was then heated to 120°C for 1 h. After cooling to room temperature, the reaction mixture was neutralized by phosphoric acid to a pH of approximately 6.0, and then 50% (w/v) ammonium sulfate solution was added as the nitrogen source (1% of the final concentration).

### SSF of rice straw

The alkali-treated rice straw was partially hydrolyzed for 2 h at 50°C, prior to yeast inoculation, to decrease the viscosity of the suspension and to improve the reproducibility of the results. For the saccharification of rice straw, a combination of cellulase (Celluclast 1.5 L), β-glucosidase (Novozyme 188), and enzyme complex (Ultraflo L) was used for enzymatic saccharification. All enzymes were obtained from Novozymes Japan (Chiba, Japan). The enzyme activity of Celluclast 1.5 L was 80 FPU/mL; β-glucosidase with an enzyme activity of 322 CBU/mL was used to hydrolyze cellobiose. Ultraflo L contained cellulase, xylanase, pentosanase, and arabanase, with an activity of 45 FBG/g. To evaluate the SSF performance of the selected strain, *S. cerevisiae* NBRC 0224, *S. stipitis* NBRC 10063, and *C. shehatae* ATCC 22984 were used as control strains. Subsequent to a 2 h partial hydrolysis, the rice straw was cooled as quickly as possible by immersion in cold water. Cells were cultured and pelleted using centrifugation. The flask was then aseptically inoculated with 2.3 g (dry cell weight) of yeast cells, corresponding to an OD_600_ of 20, and incubated at 37°C for 72 h in a rotary shaker (150 rpm). The SSF were carried out in the same flask as the pretreatment. All experiments were performed in triplicate. The amount of glucose and xylose released from the rice straw were determined to be 33.1 g/L and 12.8 g/L, respectively, following incubation without yeasts at 50°C for 24 h. The ethanol yield was calculated as a percentage of a maximum theoretical ethanol yield of 0.51 g ethanol per gram of glucose or xylose.

## Results and discussion

### Screening of xylose-fermenting yeast

We obtained 72 yeast strains able to ferment xylose from the approximate 100 natural samples collected. The ethanol production ability of each isolated strain was determined using a rich medium with YPX medium containing 4% xylose. Among the isolated strains, 16 strains produced a significant amount of ethanol from xylose. To examine the fermentability of xylose, the time courses of ethanol concentration and xylose consumption from YPX medium at different temperatures were monitored by HPLC. We observed that one yeast strain, strain ATY839 (which was isolated from soil in Kyoto University) exhibited high xylose fermentability (data not shown).

### Temperature characterization of growth and ethanol production in strain ATY839

**Figure 1:**
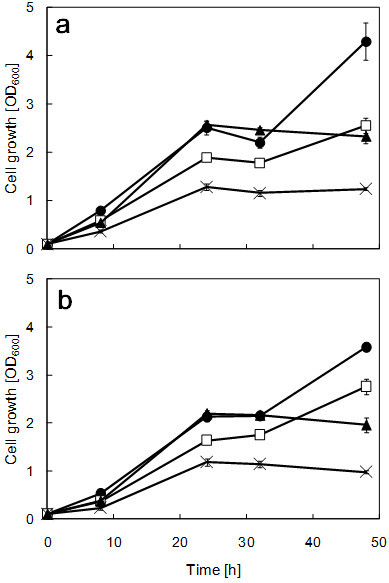
**Growth profiles of strain ATY839 in minimal medium containing 2% glucose (a) and 2% xylose (b) at 35°C (filled circle), 37°C (open square), 38°C (filled triangle), and 39°C (cross).** Data are mean ± std. dev. (error bars) of three assays.

To investigate the growth and ethanol production of strain ATY839 at different temperatures, glucose or xylose were fermented at 35, 36, 38, and 39°C. The time course analyses of cell growth in SG and SX media are shown in Figure 1a and Figure 1b, respectively. Strain ATY839 was able to grow even at 39°C in any of the carbon sources tested.

**figure 2:**
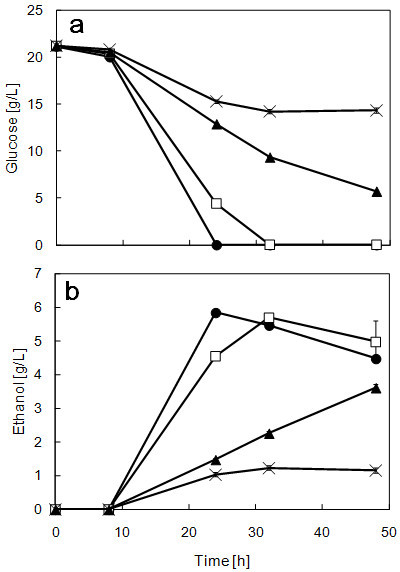
**Time course analyses of glucose consumption (a), and ethanol formation (b) in SG media using strain ATY839 at 35°C (filled circle), 37°C (open square), 38°C (filled triangle), and 39°C (cross).** Data are mean ± std. dev. (error bars) of three assays.

**Figure 3:**
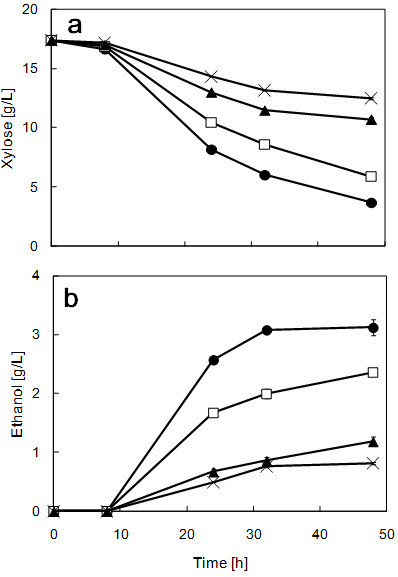
**Time course analyses of xylose consumption (a), and ethanol formation (b) in SX media using strain ATY839 at 35°C (filled circle), 37°C (open square), 38°C (filled triangle), and 39°C (cross).** Data are mean ± std. dev. (error bars) of three assays.

To determine the effects of temperature on fermentation by strain ATY839, the changes in glucose and ethanol concentrations with SG media were measured (Figure 2). As the temperature increased, glucose consumption decreased (Figure 2a). Strain ATY839 completely consumed glucose below 37°C. However, glucose consumption was not complete, above 38°C, until 48 h. At 37°C, strain ATY839 produced approximately the same amount of ethanol as at 35°C (Figure 2b). For these reasons, it is possible to say that strain ATY839 is able to maintain good glucose fermentability up to 37°C. The results of xylose and ethanol concentrations with SX media are shown in Figure 3. Sugar utilization patterns are greatly influenced by the type of sugar used as the carbon source. It is known that xylose utilization is slower compared with glucose. Moreover, the ethanol production rate of xylose-fermenting yeasts in a medium containing xylose as the sole carbon source is about half that when in a medium containing glucose as the sole carbon source ( [Grootjen et al. 1990]; [Ligthelm et al. 1988]). Therefore, xylose was not completely consumed at every temperature within 48 h. In the case of glucose fermentation, the maximum ethanol concentration at 35°C and 37°C were approximately same (Figure 2b); on the other hand, in the case of xylose fermentation, a high ethanol production level was maintained at 37°C, although the ethanol production level was a little decreased, compared with ethanol production at 35°C (approx. 80%) (Figure 3b). The data suggests that the maximum ethanol production temperature varies with the carbon source; there was a threshold of xylose-to-ethanol conversion ability between 37°C to 38°C, as found with glucose. For these reasons, it can be stated that 37°C is the upper limit of practical fermentation of strain ATY839; however, it can grow at 39°C (Figure 1). It is possible to say that strain ATY839 has a high potential for ethanol production from glucose and xylose.

It is speculated that strain ATY839 possesses defense mechanisms against and/or adaptation to higher temperatures, such as the adjustment of membrane composition and accumulation of heat shock proteins and/or trehalose ( [Devirgilio et al. 1994]; [Zhao & Bai 2009]). As the xylose-fermenting ability is weakened at elevated temperature, suitable conditions such as pH, aeration, cell density, and nutrient concentrations will need to be determined in detail.

### Taxonomic identification of strain ATY839

For the phylogenetic analysis of strain ATY839, 26S rDNA sequencing was performed and the assimilation potential of various sugars was tested (Table 1). The sequence of 26S rDNA of strain ATY839 showed 99.5% identity to that of *Candida shehatae* CBS 4704. The sequence has been recorded in the DDBJ database under the accession number AF178049. The sugar assimilation test of strain ATY839 gave results similar to those for *C. shehatae* CBS 5813 ( [Kurtzman et al. 2010]). For these reasons, we regard strain ATY839 as belonging to *C. shehatae* and it is one of the most tolerant strains to high temperature within *C. shehatae*.

**Table 1:** **Assimilation patterns of various sugars of strain ATY839**

D-Glucose	+	D-Sorbitol	+
Glycerol	+	α-Methyl-D-glucoside	+
2-Keto-D-gluconic acid hemicalcium salt	+	*N*-Acethyl-glucosamine	+
		D-Cellobiose	+
L-Arabinose	**−**	D-Lactose	w
D-Xylose	+	D-Maltose	+
Adonitol	+	D-Saccharose	+
Xylitol	w	D-Trehalose	+
D-Galactose	+	D-Melezitose	+
Inositol	w	D-Raffinose	**−**

### SSF using alkali-treated rice straw at 37°C

**Figure 4:**
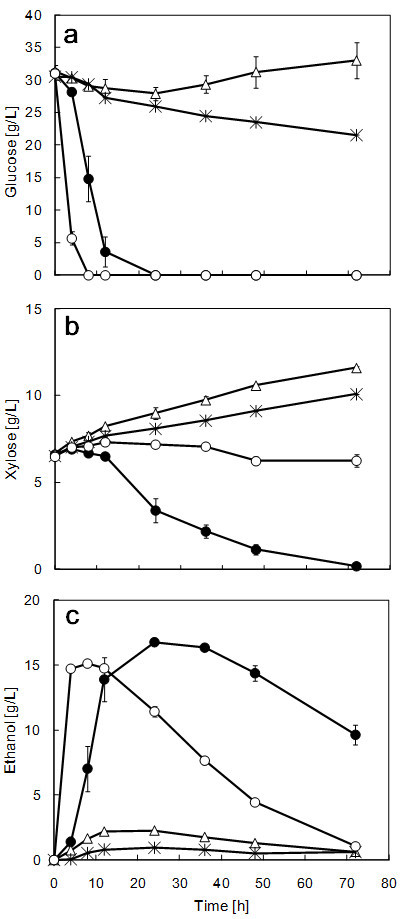
**Time course analyses of glucose consumption (a), xylose consumption (b), and ethanol formation (c) in the SSF process of rice straw using*S. cerevisiae*NBRC 0224 (open circle),*S. stipitis*NBRC 10063 (asterisk),*C. shehatae*ATCC 22984 (open triangle), and strain ATY839 (filled circle) at 37°C.** Data are mean ± std. dev. (error bars) of three assays.

Strain ATY839 showed superior fermentative performance at elevated temperature, as can be seen in Figures 1, 2 and 3. Strain ATY839 was found to be more suitable for lignocellulosic biomass fermentation than *S. cerevisiae*, as xylose is the major constituent; accordingly, we demonstrated this suitability by performing SSF using rice straw as a substrate at 37°C. Data on the ethanol production and sugar consumption in SSF of alkali-pretreated rice straw are shown in Figure 4. Both *S. stipitis* NBRC 10063 and *C. shehatae* ATCC 22984 produced hardly any ethanol. In contrast, *S. cerevisiae* NBRC 0224 and strain ATY839 produced substantial amounts of ethanol from glucose and xylose derived from rice straw. This result was in agreement with those obtained in the experiments using minimal medium. The fermentation by *S. cerevisiae* NBRC 0224 was almost complete after 8 h, with an ethanol concentration of 15.1 g/L. The maximum theoretical ethanol yield of *S. cerevisiae* NBRC 0224 at 8 h was 64.5%. After 8 h fermentation, the ethanol concentration decreased which may be attributable to the assimilation of yeast cells. During SSF, using strain ATY839, the ethanol concentration reached 16.8 g/L (71.6% of the maximum theoretical ethanol yield) at 24 h; after 24 h, the ethanol concentration in the ATY839 strain cultures was slightly decreased despite the presence of xylose. The reduction of ethanol in strain ATY839 cultures may be attributable to excess aeration. It has been reported that the aeration rate was one of the most important parameters in attaining maximum ethanol concentration with xylose-fermenting yeasts ( [Sanchez et al. 1997]). This phenomenon is possibly due to the change of metabolism, but metabolic rationale is another factor to be considered and will be the focus of our future studies. Strain ATY839 completely consumed xylose within 72 h under high-temperature conditions. Although it is difficult to control the dissolved oxygen level in the SSF of rice straw, the ethanol-producing ability should be assessed in our strains under optimum conditions in future work. However, this study has confirmed that strain ATY839 performed better than the other xylose-fermenting yeasts tested.

Although *S. stipitis* is a yeast exhibiting excellent xylose-fermenting ability, it did not perform well under high-temperature conditions; in contrast to strain ATY839 which produced ethanol under such conditions. Among the *C. shehatae* strains, ATCC 22984 is a well-characterized strain; Li et al. ( [Li et al. 2012]) isolated a high-ethanol-yield mutant from *C. shehatae* ATCC 22984 by UV irradiation and cultivation with a medium containing antimycin A, suggesting that the introduction of a mutation may be effective for improving *C. shehatae*. It is possible that *C. shehatae* mutants derived from strain ATY839 by a method such as that described in Li et al. ( [Li et al. 2012]) will show more suitable characteristics for bioethanol production from a lignocellulosic biomass.

In conclusion, this study provides the first report of the isolation of a natural yeast strain, designated ATY839, which converts both glucose and xylose to ethanol with high efficiency at an elevated temperature. The fermentation experiments in SX medium revealed that strain ATY839 efficiently produced ethanol from xylose (Figure 3). Excellent fermentability was observed with xylose utilization even at 37°C. To our knowledge, strain ATY839 is the most tolerant strain to heat stress among the *C. shehatae* strains. Additionally, we performed SSF with strain ATY839 and control strains, and evaluated each strain’s ability to utilize rice straw as the feedstock for bioethanol production. The results suggest that strain ATY839 is more suitable than *S. cerevisiae* or *S. stipitis* for producing ethanol from a lignocellulosic biomass. However, further investigations are required to improve the ability of strain ATY839 to produce ethanol from sugars derived from a lignocellulosic biomass. It may also be useful as a genetic resource for engineering xylose metabolism in *S. cerevisiae* in order to improve its ability to convert xylose to ethanol.
